# Self-assembled core-shell nanoparticles with embedded internal standards for SERS quantitative detection and identification of nicotine released from snus products

**DOI:** 10.3389/fchem.2024.1348423

**Published:** 2024-03-27

**Authors:** Yongfeng Tian, Lu Zhao, Xiaofeng Shen, Shanzhai Shang, Yonghua Pan, Gaofeng Dong, Wang Huo, Donglai Zhu, Xianghu Tang

**Affiliations:** ^1^ Technology Center of China Tobacco Yunnan Industrial Co., Ltd., Kunming, China; ^2^ Anhui Institute of Optics and Fine Mechanics, HFIPS, Chinese Academy of Sciences, Hefei, China; ^3^ Yunnan Academy of Tobacco Agricultural Sciences, Kunming, China; ^4^ Hongta Tobacco (Group) Co., Ltd., Yuxi, China; ^5^ Institute of Solid State Physics, Hefei Institutes of Physical Science, Chinese Academy of Sciences, Hefei, China

**Keywords:** nicotine, snus, SERS (surface enhanced Raman spectroscopy), internal standards, liquid-liquid interface

## Abstract

Surface enhanced Raman spectroscopy (SERS) is a unique analytical technique with excellent performance in terms of sensitivity, non-destructive detection and resolution. However, due to the randomness and poor repeatability of hot spot distribution, SERS quantitative analysis is still challenging. Meanwhile, snus is a type of tobacco product that can release nicotine and other components in the mouth without burning, and the rapid detection technique based on SERS can reliably evaluate the amount of nicotine released from snus, which is of great significance for understanding its characteristics and regulating its components. Herein, the strategy was proposed to solve the feasibility of SERS quantitative detection based on self-assembled core-shell nanoparticles with embedded internal standards (EIS) due to EIS signal can effectively correct SERS signal fluctuations caused by different aggregation states and measurement conditions, thus allowing reliable quantitative SERS analysis of targets with different surface affinity. By means of process control, after the Au nanoparticles (Au NPs) were modified with 4-Mercaptobenzonitrile (4-MBN) as internal standard molecules, Ag shell with a certain thickness was grown on the surface of the AuNP@4-MBN, and then the Au@4-MBN@Ag NPs were used to regulate and control the assembly of liquid-liquid interface. The high-density nano-arrays assembled at the liquid-liquid interface ensure high reproducibility as SERS substrates, and which could be used for SERS detection of nicotine released from snus products. In addition, time-mapping research shows that this method can also be used to dynamically monitor the release of nicotine. Moreover, such destruction-free evaluation of the release of nicotine from snus products opens up new perspectives for further research about the impact of nicotinoids-related health programs.

## Introduction

In recent years, due to its characteristics of high sensitivity, high resolution, rich material structure information and non-destructive detection capability, Surface enhanced Raman spectroscopy (SERS) has become a powerful characterization and analytical technique, and which has been widely used in life science, materials science and analytical chemistry (Zhou et al., 2021; Liu H. et al., 2023; Liu W. et al., 2023; Itoh et al., 2023; Kitaw et al., 2023). And we know that stable, reliable and universal quantitative detection capability is the basic condition for evaluating the maturity of a detection and analytical method. At present, a large number of SERS substrate researches focus on the control of the hot spot structure, in order to achieve the controllable assembly at the nanoscale to improve the sensitivity, but often ignore the stability and reproducibility of the substrate and the anti-interference of the detection capability (Fang et al., 2022; Visaveliya et al., 2022; Zhang et al., 2022). As a result, SERS detection still fails to provide accurate and reliable analysis data in practical applications. That is, due to the randomness and poor repeatability of hot spot distribution, SERS quantitative analysis is still challenging, thus it can only be used for qualitative detection of preliminary screening. Only when SERS detection can be extended to the field of quantitative detection, can the detection and analysis method have a broader practical prospect.

Because of this, many quantitative detection strategies have been proposed. Among these, labeling strategy is commonly used for SERS quantitative detection, including external standard quantification method and internal standard quantification method (Shen et al., 2015; Chen et al., 2016; Jiang et al., 2022; Li G. et al., 2023). In the case of the embedded internal standard (EIS) method, a fixed amount of internal standard molecules, such as 4-Mercaptopyridine (4-Mpy) (Li G. et al., 2023), 4-aminothiophenol (4-ATP) (Zhou et al., 2020), 4-methylthiobenzoic acid (4-MBA) (Lin et al., 2018), which are first labeled on the core, and then the shell growth is carried out, so as to form the nanostructure unit of the shell protecting the internal standard molecules. Meanwhile, when constructing highly sensitive SERS substrates, liquid-liquid interface assembly is considered to be an effective strategy for fabricating high-density hot spot structures (Tian et al., 2019; Ye et al., 2021; Zhao et al., 2023). However, affected by fluid fluctuations and Brownian motion, it was generally difficult to directly use the liquid-liquid interface assembly structure as SERS substrate, and more often it was transferred to the solid surface to form an assembly film (Mao et al., 2018; Lin et al., 2020; Li et al., 2022) for use, which would give rise to partial damage to the assembly structure that be caused by the complexity of the transfer process, or made it difficult to directly use the liquid-liquid interface assembly structure for *in situ* or quasi-in-situ detection. Therefore, the strategy was proposed to solve the feasibility of SERS quantitative detection based on self-assembled core-shell nanoparticles with EIS molecules due to EIS signal can effectively correct SERS signal fluctuations caused by different aggregation states and measurement conditions, thus allowing reliable quantitative SERS analysis of targets with different surface affinity.

In addition, in terms of application range, with the development of SERS technique, people expect the practical application of SERS can be extended to more other fields, such as for detection and identification of nicotine released from snus products. Nowadays, the argument that smoking, including passive smoking, is harmful to health has been generally accepted (Zhang et al., 2021; Hecht and Hatsukami, 2022; Lilly and Calvert, 2023). Since smokeless tobacco is considered less harmful than smoking, which makes many smokers who have trouble quitting switch to smokeless products (East et al., 2021; Bast et al., 2022; Shaikh et al., 2023), such as snus (Clarke et al., 2019; Tian et al., 2021; Tjora et al., 2022), which is a type of tobacco product that can release nicotine and other components in the mouth without burning, and the rapid detection technique based on SERS can reliably evaluate the amount of nicotine released from snus, which is of great significance for understanding its characteristics and regulating its components.

Herein, self-assembled core-shell nanoparticles with EIS molecules for SERS quantitative detection and identification of nicotine released from snus products were constructed. By means of process control, 4-Mercaptobenzonitrile (4-MBN) (Zeng et al., 2019; Wang et al., 2022; Li Q. et al., 2023) molecules were chosen as the internal standard molecules modified on the surface of Au nanoparticles (Au NPs) to explore a simple and feasible Ag shell coating method. Next, high density nano-assembly based on Au@4-MBN@Ag NPs was constructed using the liquid-liquid interface to obtain a large area single layer dense film. The high-density nano-arrays assembled at the liquid-liquid interface ensure high reproducibility as SERS substrates, and which could be used for SERS detection of nicotine released from snus products. EIS molecules were used to calibrate Raman signals of analytes by internal standard method to obtain linear fitting of relative signal strength of the target molecules to be measured. In addition, time-mapping research shows that this method can also be used to dynamically monitor the release of nicotine. Moreover, such destruction-free evaluation of the release of nicotine from snus products opens up new perspectives for further research about the impact of nicotinoids-related health programs.

## Experimental

### Materials

4-Mercaptobenzonitrile (4-MBN), chloroauric acid hydrate (HAuCl_4_·4H_2_O), and ascorbic acid (AA) were obtained from Shanghai Chemicals Company. Cetyltrimethylammonium chloride (CTAC) and sodium borohydride (NaBH_4_) were purchased from Sigma. Crystal violet (CV), sodium hypochlorite solution (NaClO, available chlorine 5%) and silver nitrate (AgNO_3_) were purchased from Aladdin, China. All reagents are of analytical grade and used without further purification. All experimental glassware was washed with aqua regia before use. Milli-Q deionized water (18.2 MΩ cm) was used for all preparations.

### Synthesis of Au NPs

CTAC-stabilized homogeneous Au NPs were synthesized according to the literature (Hanske et al., 2017) with a small modification. Typically, first, under gentle stirring at room temperature, 0.2 mL of a 25 mM HAuCl_4_ solution was added to 10 mL of a 0.1 M CTAC solution. And then, 0.4 mL of a 20 mM NaBH_4_ solution was quickly injected into the mixture under vigorous stirring. After several minutes, 5 mL above solution was taken out and added into 45 mL of 0.1 M CTAC solution, that is the Au seeds solution was obtained. The second step is to acquire Au NPs. Under gentle stirring at room temperature, 3.6 mL of Au seeds solution was added to 40 mL of 25 mM CTAC solution followed by 0.16 mL of a 0.1 M AA solution was added. After 10 min, under vigorous stirring, 0.4 mL of 25 mM HAuCl_4_ was quickly injected into the mixture. After the mixture was left undisturbed at room temperature for 1 h, 50 μL of NaClO and 20 μL of 25 mM HAuCl_4_ were added under gentle stirring. After standing for 12 h at room temperature, the resulting Au NPs were centrifuged and followed by redispersion in deionized water for further characterization and application.

### Fabrication of Au NPs@4-MBN

Under gentle stirring at room temperature, 0.25 mL of NaOH (1%w/w) solution was added into 20 mL of Au NPs solution. And then, 0.4 mL of a 0.1 mM 4-MBN solution was added. After standing for 4 h in water bath at 45°C, the Au NPs functionalized with 4-MBN were centrifugated and followed by redispersed to original volume in 50 mM CTAC solution for further characterization and application.

First, under gentle stirring at room temperature, 10 mL above Au NPs@4-MBN solution was taken out and 0.1 mL NaOH (1%w/w) was added into it followed by a certain volume of a 1 mM AgNO_3_ solution was added. Next, 0.5 mL of 10 mM AA solution was added. After standing for 6 h in water bath at 65°C, the resulting Au@4-MBN@Ag NPs solution was centrifuged and followed by redispersion in deionized water before use. As a comparative study, Au@Ag NPs was synthesized by the same process without 4-MBN molecules.

### Self-assembly of Au@4-MBN@Ag NPs at the liquid-liquid interface

First, 100 mL of Au@4-MBN@Ag NPs solution was centrifuged and the supernatant was discarded, then the supernatant was redispersed to the original volume in water under ultrasonic oscillation. Then it was centrifuged again and followed by redispersion in 4 mL deionized water. Next, 1 mL of Au@4-MBN@Ag NPs concentrated solution was poured into the Petri dish with 3 mL deionized water, and 0.5 mL of cyclohexane was slowly added to form the water-oil interface layer. After 0.5 mL ethanol was rapidly added to the interface, the Au@4-MBN@Ag NPs rapidly aggregate at the interface to form a thin film with metallic luster, and which could be used for SERS substrates.

### Characterization and instruments

The absorption spectra were obtained using a UV-2550 spectrophotometer. The scanning electron microscopy (SEM) images were taken by an Auriga focused ion-beam scanning electron microscopy (FIB-SEM). X-ray photo-electron spectroscopy (XPS, ESCALAB 250, Thermo-VG Scientific, United States) was used to record the elemental information. Transmission electron microscopy (TEM) images were obtained using a FEI Thecnai G2 F20S-TWIN. Raman spectra were performed on a LabRAM HR800 confocal microscope Raman system (Horiba Jobin Yvon) using a He–Ne laser operating at 632.8 nm. The laser power was approximately 1 mW and the laser beam was focused on the sample using a ×10 LMPLFLN microscope objective (numerical aperture, NA = 0.25; working distance, WD = 10.6 mm).

## Results and discussion

**SCHEME 1:**
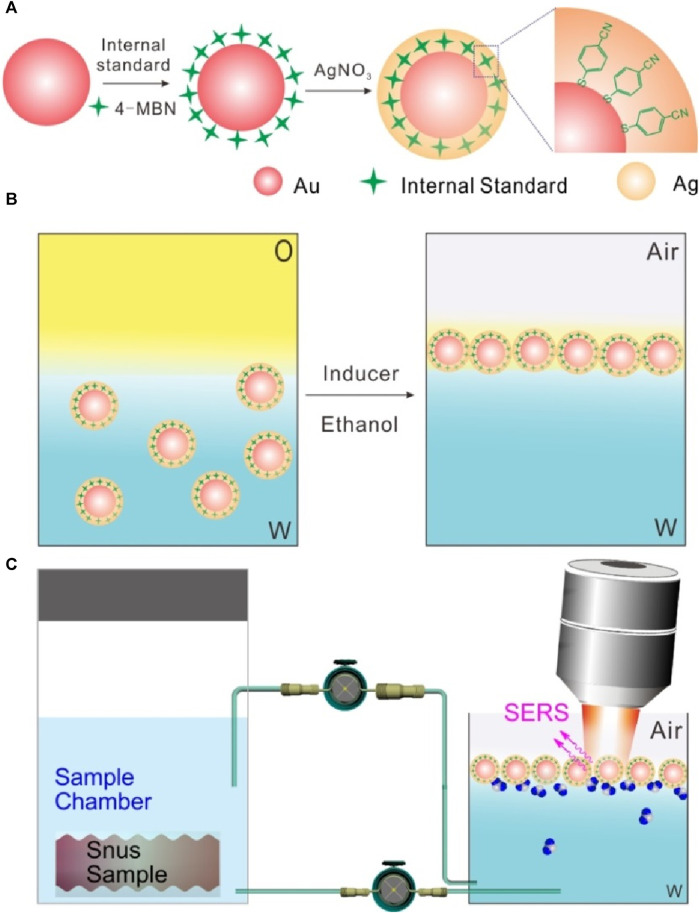
**(A)** Schematic illustration of the synthesis of Au@4-MBN@Ag NPs. **(B)** Illustration of self-assembly of Au@4-MBN@Ag NPs at the cyclohexane-H_2_O interface. **(C)** Quasi-synchronous extraction and SERS detection of nicotine release from the snus pouch based on liquid-liquid interface self-assembling Au@4-MBN@Ag NPs.

Scheme 1A displays a schematic illustration of the synthesis of Au@4-MBN@Ag NPs. According to the principle of screening internal standard signals, 4-MBN, a molecule with high Raman activity, chemical coupling with AuNP core and characteristic peaks in silent signal regions (Zeng et al., 2018; Zeng et al., 2019), was selected as the internal standard. After Ag shell with a certain thickness was grown on the surface of the Au@4-MBN NPs, and then the Au@4-MBN@Ag NPs were used to regulate and control the assembly of liquid-liquid interface, as it was illustrated in Scheme 1B, with the addition of ethanol, which can rapidly reduce the energy at the water-oil interface, and the reduction of interface energy is the main driving force for interface assembly. The high-density nano-arrays assembled at the liquid-liquid interface ensure high reproducibility as SERS substrates, and Scheme 1C shows a schematic illustration of the quasi-synchronous extraction and SERS detection of nicotine release from the snus pouch based on liquid-liquid interface self-assembling Au@4-MBN@Ag NPs.

**FIGURE 1:**
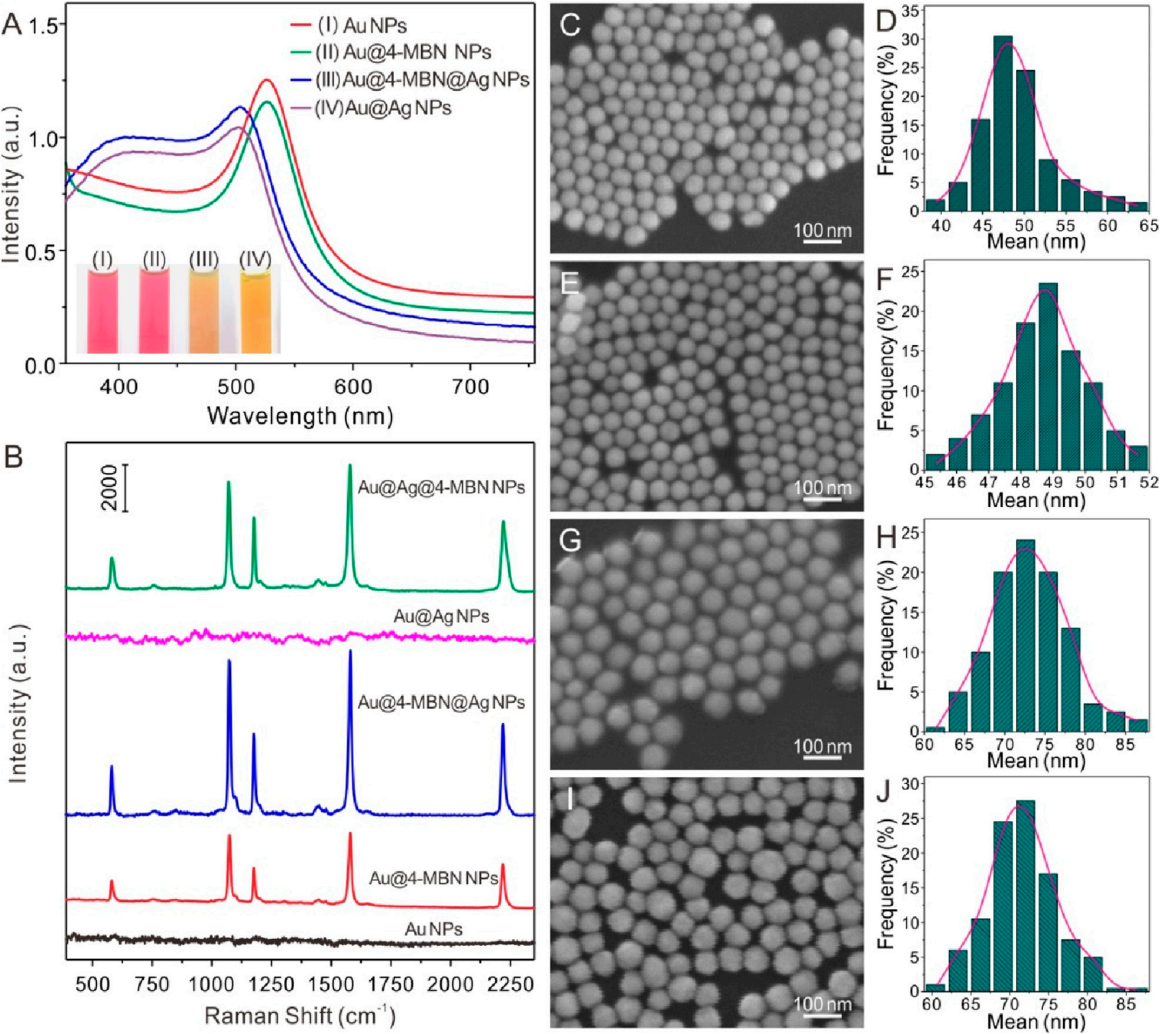
**(A)** UV−vis absorption spectra of Au NPs, Au@4-MBN NPs, Au@4-MBN@Ag NPs and Au@Ag NPs (inset: digital photos of the corresponding samples). **(B)** Typical Raman spectra of Au NPs (without 4-MBN), Au@4-MBN NPs, Au@4-MBN@Ag NPs, Au@Ag NPs (without 4-MBN), and Au@Ag NPs with 4-MBN molecules; **(C–J)** SEM and corresponding size distributions of Au NPs, Au@4-MBN NPs, Au@4-MBN@Ag NPs and Au@Ag NPs.

Figure 1A indicates the optical properties of the core of Au NPs, Au@4-MBN NPs, Au@4-MBN@Ag NPs and Au@Ag NPs was synthesized by the same process without 4-MBN molecules. It can be seen that the addition of a small number of internal standard molecules has no significant effect on the position and shape of the absorption peak. And both of the UV−vis absorption spectra of Au@4-MBN@Ag NPs and Au@Ag NPs exhibit typical optical properties of Au and Ag core-shell nanostructures (Yuan et al., 2018; Lin et al., 2019). Furthermore, SEM characterization was performed on the morphology of Au NPs, Au@4-MBN NPs, Au@4-MBN@Ag NPs and Au@Ag NPs, as shown in Figures 1C–J. Meanwhile, the sizes of various nanoparticles were also analyzed statistically. Figures 1C, D; Figure 1E, F showed relatively uniform spherical structures with average diameters about 48.3 ± 3.6 nm and 48.7 ± 1.7 nm, respectively, which indicated that modification of 4-MBN molecules almost had no significant impact on the morphology and size distribution of the Au NPs. As can be seen from Figures 1G, I, relatively uniform nanoparticles could still be obtained with the growth of Ag shell on the surface of Au NPs, but the size of particles increases significantly. From Figures 1H, J, the average size of Au@4-MBN@Ag NPs and Au@Ag NPs reached 73.1 ± 4.9 nm and 71.6 ± 4.6 nm, respectively, which indicates successful shell growth on both Au@4-MBN NPs and Au NPs surfaces, respectively.

Meanwhile, Raman spectra of Au NPs (without 4-MBN), Au@4-MBN NPs and Au@4-MBN@Ag NPs were carried out. It can be seen from Figure 1B, the background signal of Au NPs without 4-MBN was low and the overall baseline was flat, while the 4-MBN signal was clearly observed after modifying the molecules on Au NPs surface. Interestingly, when Au@4-MBN@Ag NPs was obtained, a very strong 4-MBN signal was obtained under the same detection conditions. This may be attributed to the synergistic enhancement of Raman signals of EIS molecules by the Au and Ag heterostructures (Langer et al., 2020). As a control, Raman spectra of Au@Ag NPs (without 4-MBN), and Au@Ag NPs with 4-MBN molecules were also performed. From Figure 1B, it can be seem that the background signal of Au@Ag NPs without 4-MBN was low, while the 4-MBN signal was clearly observed from the SERS substrates of Au@Ag NPs with 4-MBN molecules, which indirectly proves that Au@EIS@Ag NPs werd indeed embedded with 4-MBN molecules.

**FIGURE 2:**
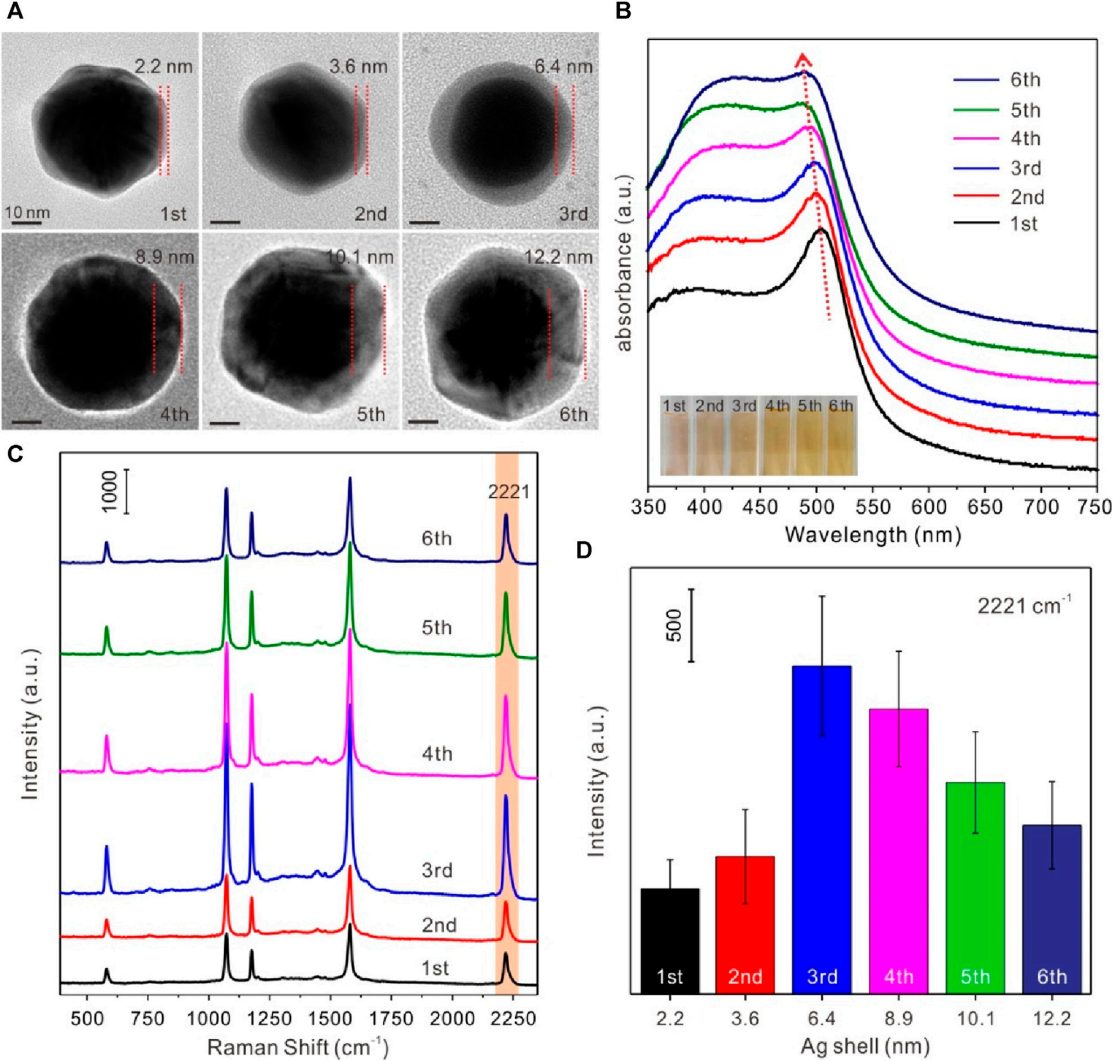
**(A)** TEM images of Au@4-MBN@Ag NPs with Ag shell thickness of 2.2, 3.6, 6.4, 8.9, 10.1 and 12.2 nm; **(B)** UV−vis absorption spectra (inset: digital photos) of Au@4-MBN@Ag NPs with different shell thicknesses; **(C)** Raman spectra of Au@4-MBN@Ag NPs with different shell thicknesses excited with 633 nm laser; **(D)** Raman intensity of different shell thicknesses of Au@4-MBN@Ag NPs at 2221 cm^−1^.

Considering the effect of Ag shell on SERS activity of Au@4-MBN@Ag NPs, the thickness of Ag shell was regulated and the relationship between its structure and SERS effect was studied. A series of Au@4-MBN@Ag NPs with different shell thickness were obtained by adjusting the volume of AgNO_3_ solution. Typically, in the experiment, after 10 mL Au NPs@4-MBN solution was taken out and a mixture was formed with 0.1 mL NaOH (1%w/w), a series of volumes of 0.6, 0.8, 1.0, 1.2, 1.4, and 1.6 mL of AgNO_3_ solution were then added, respectively. The more AgNO_3_ solution added, the thicker Ag shell obtained, and therefore the larger size of the nanoparticles formed, as can be seen in Figure 2A. It can be seen from the TEM images that the final synthesized particles were core-shell structures. In addition, the shell thickness of each type particle was measured with values of 2.2, 3.6, 6.4, 8.9, 10.1, and 12.2 nm, respectively. UV−vis absorption spectra of Au@4-MBN@Ag NPs with different shell thicknesses can be seen in Figure 2B. As the thickness of the Ag shell increases from 2.2 to 12.2 nm, the LSPR peak of Au@4-MBN@Ag NPs shows a blue shift from 505 nm to 486 nm, which is very consistent with the traditional Mie scattering theory and dielectric data (Wang X. et al., 2016; Cong et al., 2020).

The most critical point of this study was that after the Ag shell has been grown on the outer layer of the Au@4-MBN NPs, the signal of EIS based on 4-MBN molecules could still appears, so as to ensure that the EIS molecules would not be damaged during the growth of the shell. Moreover, the thickness of the shell should be moderate, and the Raman peak of 4-MBN can be clearly identified under the excitation of Laser. Therefore, Raman acquisition based on a series of Au@4-MBN@Ag NPs was conducted, as shown in Figure 2C. From the results, it can be seen that all Au@4-MBN@Ag NPs showed stronger Raman enhancement peaks of 4-MBN and the characteristic peak at 2221 cm^-1^, which is tentatively assigned to the nitrile (CN) stretch of 4-MBN in silent signal region (Wang S. et al., 2016; Sorenson et al., 2017), is particularly significant. Among them, when the thickness of the Ag shell was 6.4 nm, the strongest Raman signal intensity was shown clearly in Figures 2C, D. There may be several reasons for this phenomenon. First, when the Ag shell reaches a certain thickness, the synergistic enhancement of Raman signals of EIS molecules by Au and Ag heterostructures becomes significant. Second, when the thickness of the Ag shell increases, the laser excitation intensity to 4-MBN molecules would be decreased due to the damping of the shell. Or say, the thicker shell obstructs the output of the SERS signal of the EIS molecules. Third, the larger shell may affect its electromagnetic coupling with the Au core and reduce the effect of electromagnetic field enhancement. Therefore, Au-Ag core-shell nanoparticles with 6–7 nm shell thickness were selected as the SERS substrate for detection.

**FIGURE 3:**
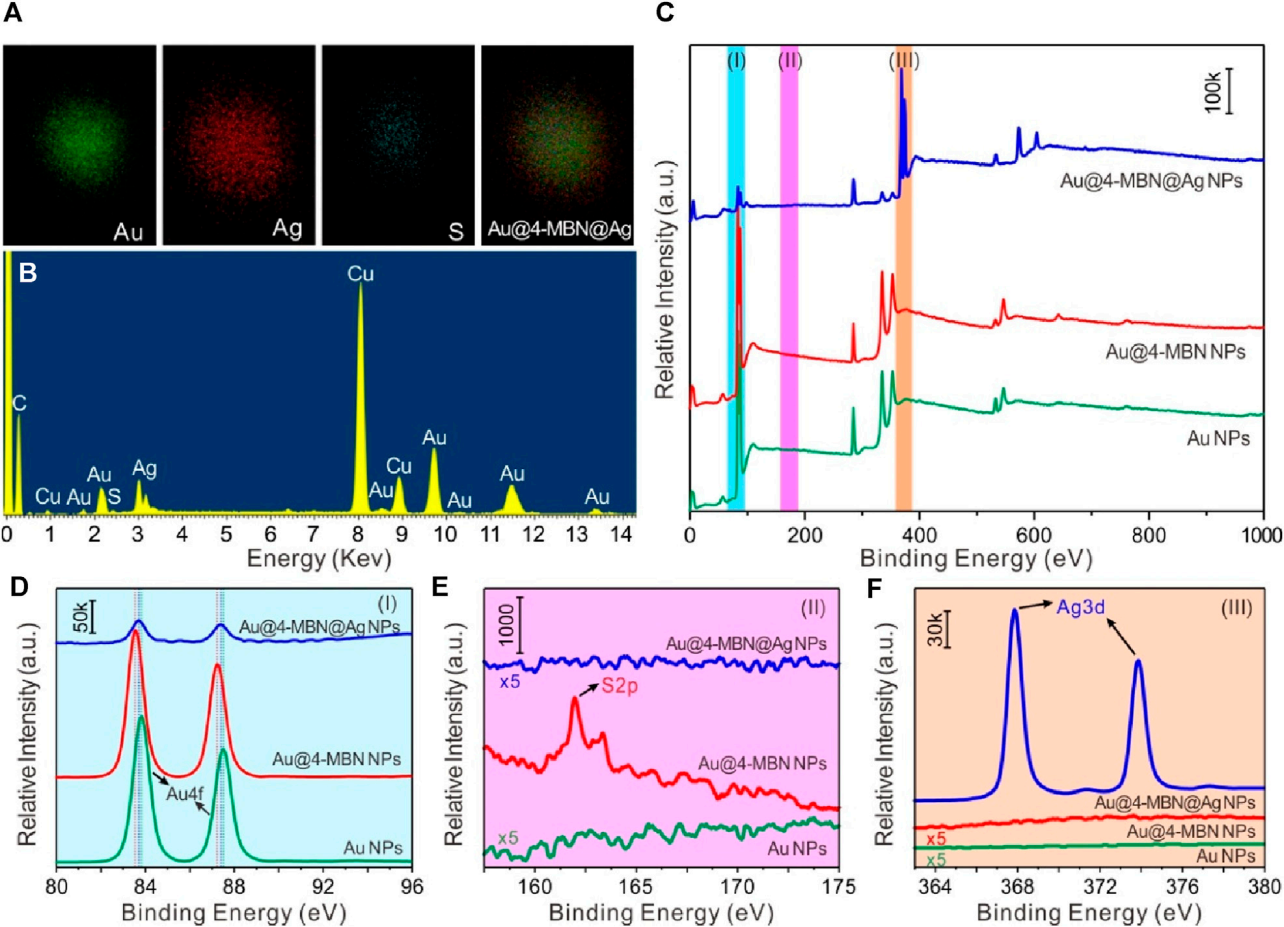
**(A)** Typical elemental mappings and **(B)** EDS spectrum of Au@4-MBN@Ag NPs with Ag shell thickness of 6.4 nm. **(C)** XPS spectra of Au NPs, Au@4-MBN NPs, and Au@4-MBN@Ag NPs, **(D–F)** High-resolution Au 4f, S 2p, and Ag 3d spectra corresponding to the XPS spectra represented by (I) azure, (II) magenta, and (III) tangerine bars in **(C)**, respectively.

In order to further explore the composition of Au@4-MBN@Ag NPs, EDS spectrum was used for elemental analysis of the sample while TEM characterization was performed, as shown in Figure 3, which is corresponding to the Au@4-MBN@Ag NPs sample with about 6.4 nm Ag shell thickness. The EDS spectra confirmed that the sample was mainly composed of Au and Ag elements, while a small amount of S element was derived from EIS based on 4-MBN molecules. It should be noted here that the Cu element shown in the EDS spectrum was derived from the copper network, and the C element was mainly derived from the carbon film of the copper network, and a small part may also be derived from EIS molecules. Through the face scanning energy spectrum analysis of Au La1 Ag La1 and S Ka1, we further studied the element distribution of Au@4-MBN@Ag NPs samples, as shown in Figure 3A. Since individual particle was selected for scanning, the distribution of elements can be seen that the distribution of Au element is obviously smaller than that of Ag element, which is consistent with the structure of the outer Ag shell with Au NPs as the core. The circular distribution of the S element indicates that it is distributed throughout the structure, and the contour range is smaller than that of the Ag element, that is, the core-shell particles are successfully embedded with the internal standard molecular layer. Meanwhile, in order to obtain further information about the surface composition of the samples, XPS studies were carried out, as shown in Figure 3C. From detailed XPS study results in Figure 3D, it can be seen that there are some slight differences in the spectral peaks of the Au 4f peaks of Au NPs, Au@4-MBN NPs, and Au@4-MBN@Ag NPs due to the changes of the chemical environment (Wang et al., 2021) of the surface or interfacial of Au NPs. Comparing the three curves in Figure 3E, the S 2p peak at 162 eV was attributed to the Au–S bond (Elliott et al., 2015) formed by SH- and the Au surface, indicating that a 4-MBN molecular layer modified to the Au NPs surface. Moreover, the S 2p peak disappeared with the appearance of Ag 3d peak (as can be seen in Figure 3F) after the formation of the core–shell structure, proving that there were no 4-MBN molecules on Au@4-MBN@Ag NPs surface. Combined with the preceding results of Raman spectra, it can be concluded that the 4-MBN was indeed bound to the Au surface and there is no 4-MBN on the outer surface of Au@4-MBN@Ag NPs, which further indicated that 4-MBN molecules were embedded between the Au core and Ag shell and could be used for quantitative SERS analysis studies.

**FIGURE 4:**
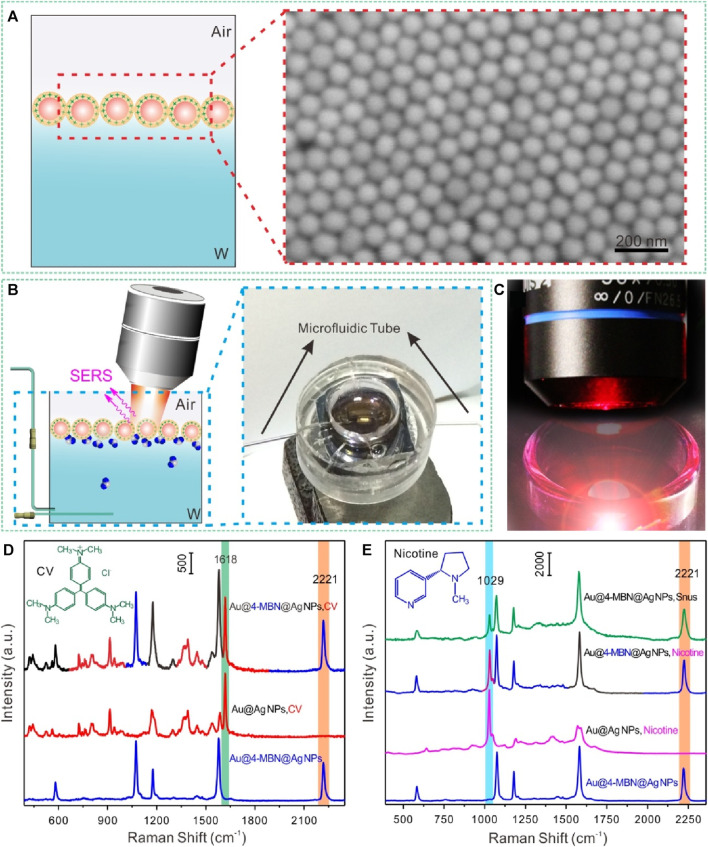
**(A)** Illustration of self-assembly at the cyclohexane-H_2_O interface of Au@4-MBN@Ag and typical SEM characterization image. **(B)** Illustration of *in situ* SERS detection based on liquid-liquid interface self-assembling Au@4-MBN@Ag NPs in reaction cell and digital photo. **(C)** Photo of SERS detection. **(D)** Typical Raman spectra of Au@4-MBN@Ag NPs, CV with Au@Ag NPs and Au@4-MBN@Ag NPs as substrates, respectively. **(E)** Typical Raman spectra of Au@4-MBN@Ag NPs, Au@Ag NPs with nicotine standard solution, Au@4-MBN@Ag NPs with nicotine standard solution, and Au@4-MBN@Ag NPs with snus extraction solution, respectively.

For Au@4-MBN@Ag NPs substrate, as its SERS signal molecule was embedded in the nanostructured core-shell and was not susceptible to external interference, the shell could be used as the enhanced surface for SERS substrate while the embedded 4-MBN molecules were used as SERS internal standard, without any competition between the EIS molecules and the analytes to be detected. That is, the Raman signal of EIS molecules and the analytes can be obtained simultaneously. To obtain quantitative information of the interface, Au@4-MBN@Ag NPs needs to be assembled at the liquid-liquid interface, which ensures the signal sensitivity of the analytes and further uses the calibration of signals from EIS molecules to reduce external interference. The schematic illustration of self-assembly at the cyclohexane-H_2_O interface of Au@4-MBN@Ag NPs and typical SEM characterization image are shown in Figure 4A. By transferring the assembled nanoparticles at the liquid-liquid interface to the surface of the wafer for SEM characterization, it can be seen that the nanoparticles form a compact monolayer assembled layer, which is conducive to improving SERS detection sensitivity. Figure 4B illustrated the *in situ* SERS detection platform based on liquid-liquid interface self-assembling Au@4-MBN@Ag NPs in reaction cell, the photo of SERS detection can be seen in Figure 4C. In order to investigate whether Au@4-MBN@Ag NPs could be used for SERS quantitative detection, CV and nicotine standard solution were used as the model probe molecules. From the SERS characteristic peaks of 4-MBN molecule in Figures 4D, E, we can see that the SERS spectra of CV and nicotine molecules are easy to distinguish from it and have obvious non-overlapping peaks. Therefore, 4-MBN is suitable for EIS molecules to calibrate the SERS signals of CV and nicotine molecules.

**FIGURE 5:**
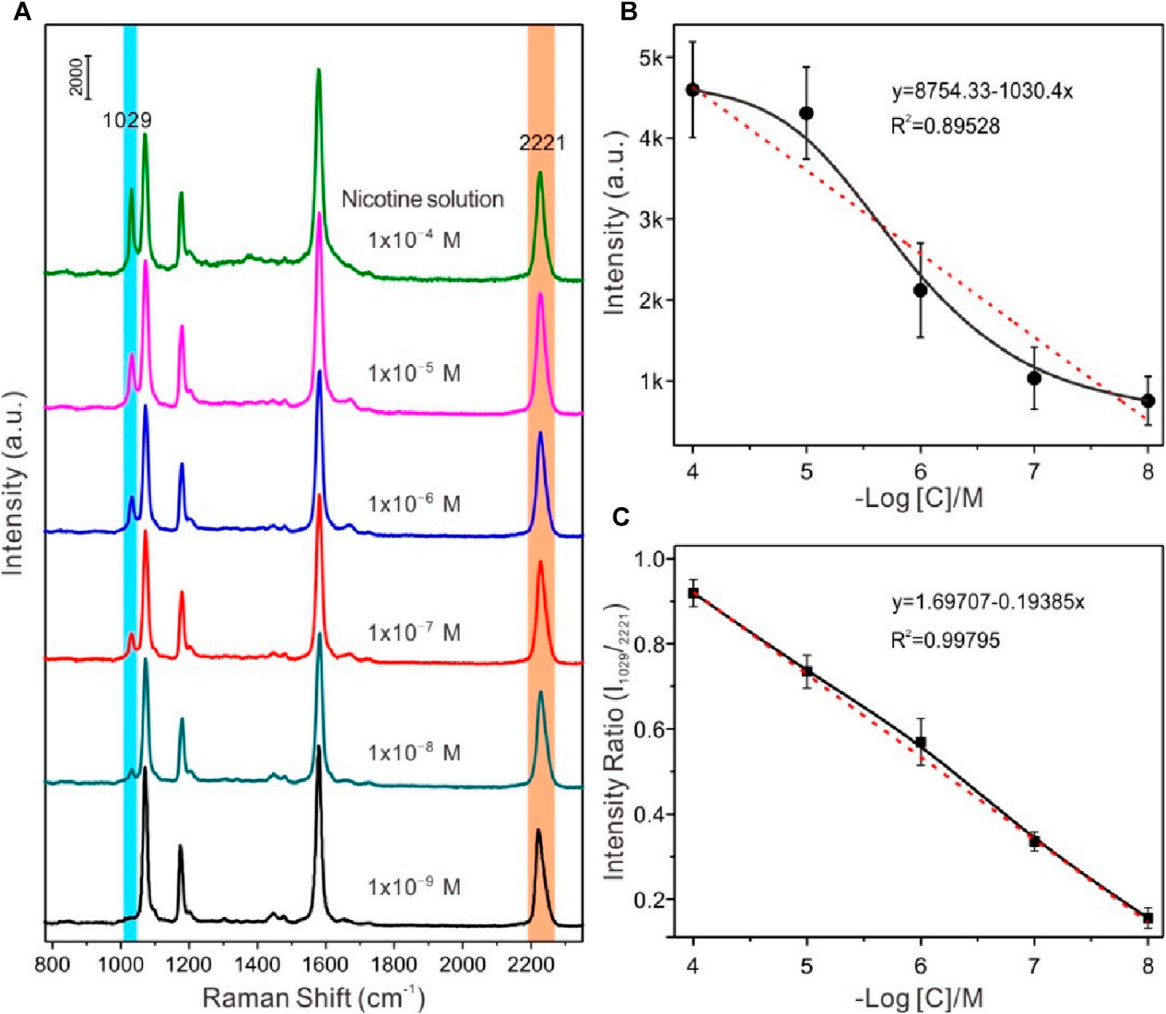
**(A)** Series of SERS spectra of nicotine in water at different concentrations with liquid-liquid interface self-assembling Au@4-MBN@Ag NPs as the SERS substrates, respectively. **(B)** and **(C)** Plot of the SERS intensities at 1029 cm^-1^ *versus* the snus concentration over a large concentration range.

In order to explore the relationship between the concentration of the analytes and SERS signal intensities, the negative value of the spectral peak intensity and concentration logarithm at 1029 cm^-1^ for data fitting was selected, in which the negative value of the concentration logarithm was taken as the horizontal coordinate, and the SERS spectral peak intensity value was taken as the ordinate. Figure 5A exhibited a series of SERS spectra of nicotine in water at different concentrations with liquid-liquid interface self-assembling Au@4-MBN@Ag NPs as the SERS substrates, respectively. The analyte molecules exhibited a dominant peak at 1029 cm^−1^, a shoulder at 1049 cm^−1^, and other smaller peaks. Compared with the normal Raman spectrum of nicotine from our previous research and ref. (Li et al., 2021; Tian et al., 2021), the peaks at 1029 cm^−1^ and 1049 cm^−1^ are tentatively assigned to the symmetrical breathing and trigonal ring deformation of the pyridine moiety. It can also be seen from Figure 5A, the detection ability of the substrate for nicotine solution can reach the level of 1 × 10^−8^ M. Moreover, as can be seen from Figure 5B, due to the interference of various factors, the relationship between the logarithm of SERS signal intensity and concentration of nicotine molecules has a wide range of fluctuations. At the same time, the signal of the EIS, 4-MBN molecules also produced irregular fluctuations in a series of detection processes. From the SERS effect characterization of Au@4-MBN@Ag described above, it was already known that the great fluctuation of SERS signal was related to the non-uniform distribution of “hot spots” on the surface of the material in the two-dimensional domain, which was also the reason for the low reproducibility of most SERS substrates used for detection. If the ratio of spectral peak intensity at 1029 cm^-1^ of nicotine molecules to be detected and 2221 cm^-1^ of 4-MBN are used to fit the negative value of concentration logarithm, it can be found that the obtained relative intensity shows a good linear relationship with the concentration of the analytes in the mixed liquid added by drops, as shown in Figure 5C. Thus, within a certain concentration range of the substance to be detected, the EIS moleculeS in Au@4-MBN@Ag could provide effective signal feedback when the substrate was used for detection, that is, Au@4-MBN@Ag NPs could be used for SERS quantitative detection and analysis.

**FIGURE 6:**
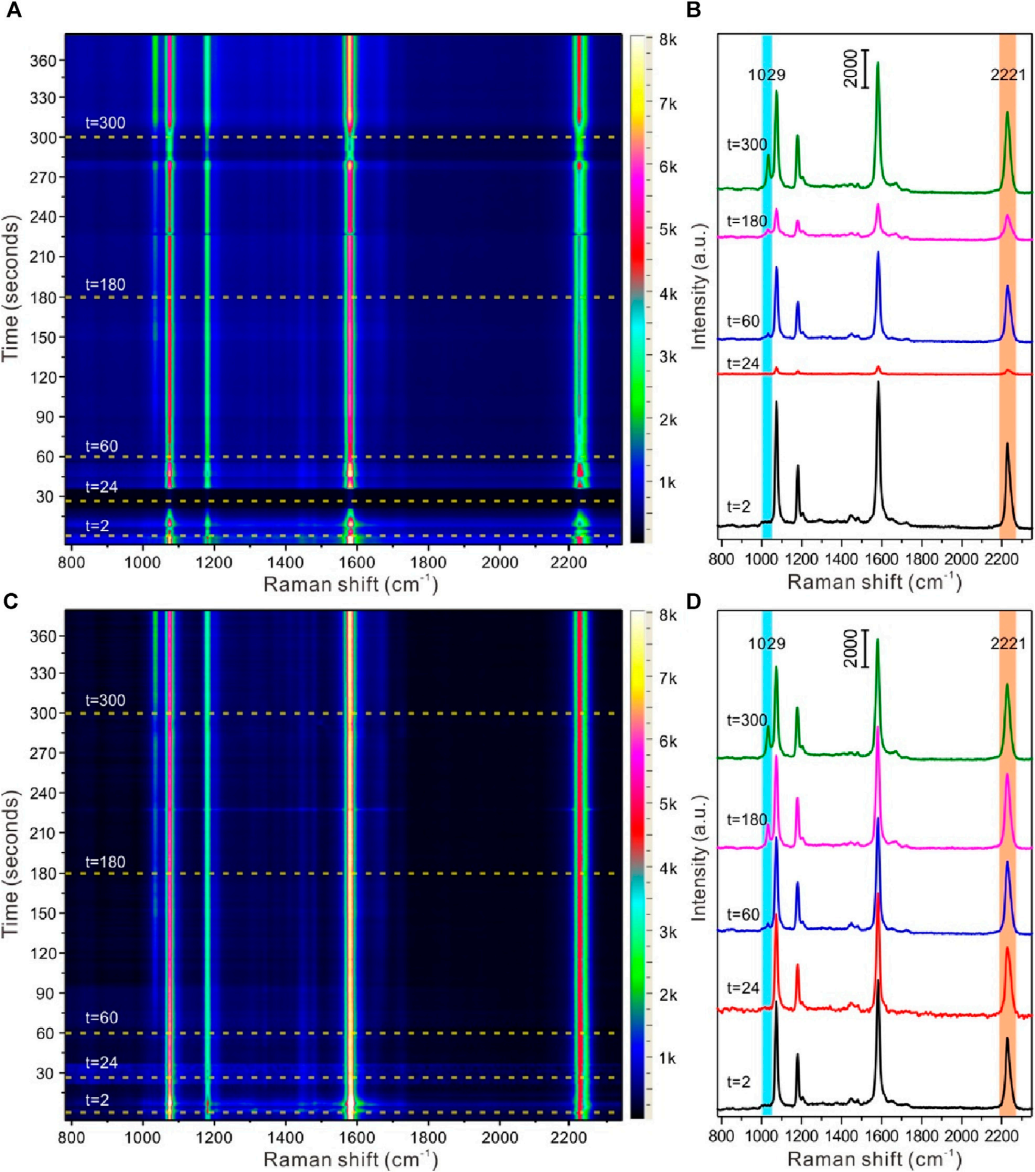
SERS detection of nicotine release from snus based on liquid-liquid interface self-assembling Au@4-MBN@Ag NPs in reaction cell. **(A, B)** Time-resolution SERS mapping and typical SERS spectra before calibration based on EIS signals; **(C, D)** Time-resolution SERS mapping and typical SERS spectra after calibration based on EIS signals.

Based on the above discussion, furthermore, the strategy of self-assembled core-shell nanoparticles with EIS molecules for SERS quantitative detection and identification of nicotine released from snus products was carried out. Figure 6 displayed the time-dependent SERS detection of nicotine release from snus based on liquid-liquid interface self-assembling Au@4-MBN@Ag NPs in reaction cell before and after calibration based on EIS signals. The snus product and the homemade snus pouch can be seen in Supplementary Figure S1. As can be seen from Figures 6A, B, after the liquid-liquid interface assembly structure was formed and the microfluidic tube between the *in situ* cell and the snus extraction cell was connected without extraction solution from snus, all Raman signals come from EIS molecules. Meanwhile, due to the interference of microfluidic flow at the liquid-liquid interface, the Raman signal intensity of the system fluctuates greatly within tens of seconds after the equilibrium of the liquid-liquid interface was re-established. After that, the fluid tends to be relatively stable, and then the SERS signal of nicotine molecules can be observed while extraction solution from snus fully dispersed in the *in situ* detection cell. However, due to the interference of many factors such as Brownian motion and liquid flow in the liquid system, the focusing of the laser spot on the surface of the assembly structure at the liquid-liquid interface was keep fluctuating, so there was no specific variation rules from the point of view of the SERS intensity changes based on the signal of nicotine molecules. After the detection signal was calibrated based on the EIS molecules, Raman signal from 4-MBN, as shown in Figures 6C, D, it could be seen that the Raman signal of nicotine molecules could be clearly identified in the SERS spectrum of snus extraction. The Raman signal of nicotine molecules was significantly enhanced with the extension of time. This also indicates that the strategy based on liquid-liquid interface self-assembling Au@4-MBN@Ag NPs in reaction cell can be used to dynamically SERS monitor the nicotine release from snus.

## Conclusion

In this work, a type of core-shell structure Au@4-MBN@Ag NPs with 4-MBN as the EIS molecules was designed and fabricated for SERS quantitative detection and identification of nicotine released from snus products. Through the preparation and detailed structure and property characterization of Au@4-MBN@Ag NPs, the SERS signal of internal standard molecule 4-MBN was the strongest when the thickness of Ag shell was 6–8 nm, which was suitable for the subsequent further experiment. The liquid-liquid interface assembly of the nanostructure formed a high-density nanoarray to ensure its high sensitivity as a SERS substrate. Meanwhile, the 4-MBN were used as internal standard, and relatively quantitative SERS detection could be performed. The relative concentration and SERS intensity of nicotine calibrated by EIS molecule showed an excellent linear relationship. When it was applied to the monitoring of nicotine release from snus, the calibration of SERS signals of the nicotine molecules by EIS was used to obtain a good linear fit of the relative signal intensity of the analyte. The results indicated that the strategy based on liquid-liquid interface self-assembling Au@4-MBN@Ag NPs in reaction cell can be used to dynamically SERS monitor the nicotine release from snus, which also provided a technical reference for qualitative and quantitative evaluation of nicotine release from snus by SERS method.

## Data Availability

The original contributions presented in the study are included in the article/Supplementary Material, further inquiries can be directed to the corresponding authors.
