# Supplementary material for: Self-assembled core-shell nanoparticles with embedded internal standards for SERS quantitative detection and identification of nicotine released from snus products
Source: Front Chem. 2024 Mar 27;12:1348423. doi: 10.3389/fchem.2024.1348423 (PMC11005032; doi:10.3389/fchem.2024.1348423)
Supplement: Supplementary file 1 [file DataSheet1.pdf]

Electronic Supplementary Information (ESI) *for*

Self-assembled core-shell nanoparticles with embedded internal standards for SERS quantitative detection and identification of nicotine released from snus products

Yongfeng Tian[1,2], Lu Zhao[3], Xiaofeng Shen[1], Shanzhai Shang[1], Yonghua Pan[4], Gaofeng Dong[1], Wang Huo[5], Donglai Zhu[1*], Xianghu Tang[5*]

1. Technology Center of China Tobacco Yunnan Industrial Co., Ltd, Kunming 650231, China.

*E-mail: zhudl@ynzy-tobacco.com

2. Anhui Institute of Optics and Fine Mechanics,HFIPS, Chinese Academy of Sciences, Hefei 230031, China

3. Yunnan Academy of Tobacco Agricultural Sciences, Kunming 650000, China.

4. Hongta Tobacco (Group) Co., Ltd. Yuxi 653100, China

5. Institute of Solid State Physics, HFIPS, Chinese Academy of Sciences, Hefei 230031, China.

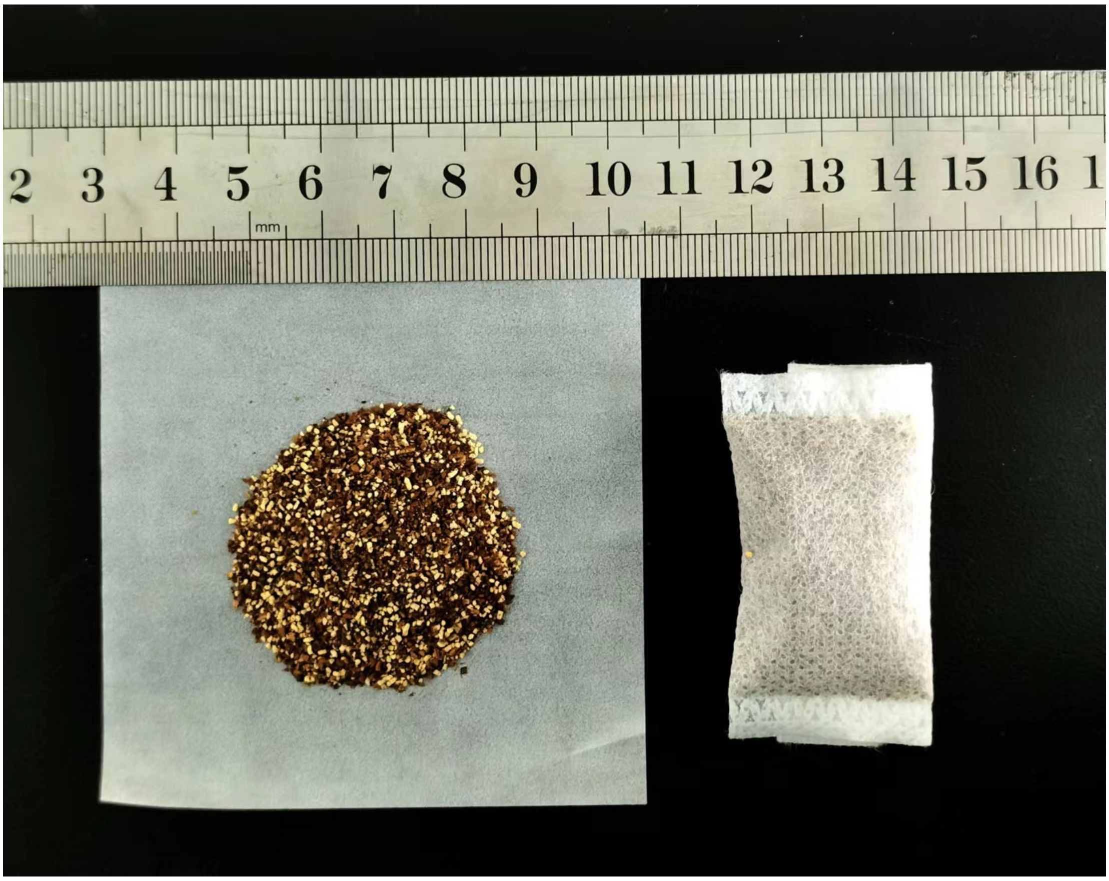

Figure S1 Digital photograph of the snus product and photograph of the homemade snus pouch.
